# Characterization of hospital-associated lineages of ampicillin-resistant *Enterococcus faecium* from clinical cases in dogs and humans

**DOI:** 10.3389/fmicb.2013.00245

**Published:** 2013-08-23

**Authors:** Cindy-Love Tremblay, Audrey Charlebois, Luke Masson, Marie Archambault

**Affiliations:** ^1^Department of Pathology and Microbiology, Faculty of Veterinary Medicine, University of Montreal, CRIPA Research GroupQuebec, QC, Canada; ^2^Biotechnology Research Institute, National Research Council of CanadaMontreal, QC, Canada

**Keywords:** *Enterococcus faecium*, clinical cases, antibiotic resistance, virulence, plasmid, hospital-associated, community-associated, MLST

## Abstract

Ampicillin-resistant *Enterococcus faecium* (ARE) has rapidly emerged worldwide and is one of the most important nosocomial pathogens. However, very few reports are available on ARE isolates from canine clinical cases. The objective of this study was to characterize ARE strains of canine clinical origin from a veterinary teaching hospital in Canada and to compare them with human strains. Ten ARE strains from dogs and humans were characterized by multilocus sequence typing (MLST), pulsed field gel electrophoresis (PFGE), antibiotic susceptibility and biofilm activities, presence of *rep*-families, CRISPR-*cas* and putative virulence genes. All ARE strains (*n* = 10) were resistant to ciprofloxacin and lincomycin. Resistances to tetracycline (*n* = 6), macrolides (*n* = 6), and to high concentrations of gentamicin, kanamycin and streptomycin (*n* = 5) were also observed. Canine ARE isolates were found to be susceptible to vancomycin whereas resistance to this antibiotic was observed in human strains. Ampicillin resistance was linked to PBP5 showing mutations at 25 amino acid positions. Fluoroquinolone resistance was attributable to ParC, GyrA, and GyrB mutations. Data demonstrated that all canine ARE were *acm* (collagen binding protein)-positive and that most harbored the *efaA_fm_* gene, encoding for a cell wall adhesin. Biofilm formation was observed in two human strains but not in canine strains. Two to five *rep*-families were observed per strain but no CRISPR sequences were found. A total of six STs (1, 18, 65, 202, 205, and 803) were found with one belonging to a new ST (ST803). These STs were identical or closely related to human hospital-associated lineages. This report describes for the first time the characterization of canine ARE hospital-associated strains in Canada and also supports the importance of prudent antibiotic use in veterinary medicine to avoid zoonotic spread of canine ARE.

## Introduction

Multiresistant *Enterococcus faecium* has become one of the most important nosocomial pathogens, causing increasing numbers of nosocomial infections worldwide (Willems et al., 2012). They now represent up to one-third of enterococcal infections (Willems and Van Schaik, 2009). A multilocus sequence typing (MLST) study of *E. faecium* isolates revealed the existence of a distinct genetic subpopulation associated with nosocomial infections which was named clonal complex 17 (CC17) (Willems et al., 2011). Recently, other studies (Van Schaik and Willems, 2010; Van Schaik et al., 2010; De Regt et al., 2012; Willems et al., 2012) have indicated that these hospital-associated *E. faecium* (HA *E. faecium*) did not evolve from a single founder [i.e., sequence type (ST) 17] as there are now several founders identified. More recently, a Bayesian population genetic analysis revealed that HA *E. faecium* could be divided into three lineages originating from STs 17, 18 and 78, all ampicillin resistant and with variable resistance phenotypes to other antibiotics (Willems et al., 2012).

Genome-wide analyses have shown that HA ARE have a genetic repertoire distinct from *E. faecium* strains that asymptomatically colonize the intestinal tract of both humans and animals in the community (Leavis et al., 2007; Van Schaik et al., 2010) and are found only sporadically among non-hospital isolates (Willems et al., 2011). However, previous studies around the world (Damborg et al., 2008, 2009; De Regt et al., 2008, 2012; Ossiprandi et al., 2008; Jackson et al., 2009; Kwon et al., 2012) have revealed that healthy dogs are known community reservoirs where some of these typical hospital clones seem to reside. Furthermore, HA ARE have been recovered from clinical cases of urinary tract infections in canines from the U.S. (Simjee et al., 2002), Korea (Kwon et al., 2012) and Denmark (Damborg et al., 2008) and from feces of canines leaving the intensive care unit of an American veterinary medicine teaching hospital (Ghosh et al., 2011). In general, only limited data is available on lineages and genotypic content of HA ARE from canine clinical isolates.

A recent report from Canadian human cases of HA ARE (McCracken et al., 2013) has indicated that a shift in ST has occurred after 2006 and that around that time bacteraemia rates began to rise in central and western Canada, suggesting a possible correlation. Prior to 2006, predominant types included ST154, ST16, ST17 and ST80 and after 2006, ST18, ST203, ST412 and ST584 became predominant in Canada (McCracken et al., 2013). In Canada, no data is available on ARE isolates from dogs with ST lineages causing nosocomial infections in humans. Thus, the goal of this study was to characterize canine ARE clinical isolates from Québec, Canada and to determine their STs. Also, HA ARE strains of human origin were used for comparison to assess possible genetic relationships between the two sets of strains. This study underscores the importance of canines as potential reservoirs of multi-drug resistant HA ARE.

## Materials and methods

### Bacterial isolates and identification

Ten *E. faecium* strains were used in this study (Table 1). Dog' strain isolation criteria were the presence of a relatively recent (2007–2012) clinical infection, isolation in pure culture, and PCR identification as *E. faecium* species. Based on these criteria only five canine *E. faecium* isolates were recovered over this period from the diagnostic laboratory of the Faculty of Veterinary Medicine at the University of Montreal and these were from UTI, wounds and cholangiohepatitis infections. The human isolates (*n* = 5) were provided by the “Centre de Recherche Hospitalier de l'Université Laval (CRCHUL)” and randomly chosen from a culture collection of *E. faecium* from hospitalized patients over the same period. Some of the human isolates (CCRI no. 18581, 16717 and 16354) were from colonization/surveillance studies of hospitalized patients. All isolates were identified by multiplex PCR assay using species-specific primer sets for the *ddl faecium* (*ddl* F-5′TTGAGGCAGACCAGATTGACG3′ and *ddl* R-5′TATGACAGCGACTCCGATTCC3′) identification gene as previously described (Tremblay et al., 2011). *E. faecium* HA-56038 was used as a positive control.

**Table 1:** **Characterization of *E.faecium* isolates from human and canine enterococcal infections**.

**Isolate no.**	**Host^a^**	**Sources**	**Phenotypic resistance^b^**	**Antibiotic and related resistance gene^c^**	**Virulence gene**	**Biofilm formation**	***Rep*-family**	**PFGE pattern**	**ST group**	**MLST allelic profile**						
										***atpA***	***ddl***	***gdh***	***purK***	***gyd***	***pstS***	***adk***
07-5598	D	Wound	CHL, CIP, ERY, GEN, KAN, LIN, AMP, PEN, STR, TET, TYL	*aac(6′)-Ii, tet*(M), *tet*(L), *trans, trans1*	*acm*	*–*	2, 11, 18	A1	202	1	1	1	1	1	7	1
M2971-08	D	Wound	CIP, ERY, GEN, KAN, LIN, AMP, PEN, STR, TET, TYL	*aac(6′)-Ii, aadE, aac(6′)-Ie-aph(2″)-Ia, msrC, tet*(M), *tet*(L), *tet*(O), *trans, trans1*, IS*150*	*efaA_fm_, acm*	*–*	2, 6, 11	A2	202	1	1	1	1	1	7	1
M2146-08	D	Bile	CIP, LIN, AMP, PEN, TET	*aac(6′)-Ii, tet*(M), *tet*(L), *tet*(O), *msrC, trans, trans1, IS150*	*efaA_fm_, acm*	*–*	2, 6, 11, 14	E1	803	1	1	1	1	1	57	1
M5853-09	D	Urine	CIP, LIN, AMP, PEN, TET	*aac(6′)-Ii, msrC, tet*(M), *tet*(L), *trans, trans1*	*agg, efaA_fm_, acm*	*–*	2, 6, 11, 14	E2	803	1	1	1	1	1	57	1
M20638-11	D	Urine	CIP, LIN, AMP, PEN	*aac(6′)-Ii, msrC, trans1*	*efaA_fm_, acm*	*–*	6, 11, 14	F	803	1	1	1	1	1	57	1
CCRI-18581	H	Anal	CIP, ERY, KAN, LIN, AMP, PEN, STR, TET, TYL, VAN	*aac(6′)-Ii, aadE, aph(3′)-IIIa, erm*(AM), *sat(4), erm*(B), *msrC, tet*(M), *vanA, vanH, vanR, vanS, vanX, vanY, trans, trans1*, IS*1182*, IS*150*	*esp, acm*	*–*	11, 14, 17, Unique	H	18	7	1	1	1	5	1	1
CCRI-16354	H	Rectal	CIP, LIN, AMP, PEN, VAN	*aac(6′)-Ii, msrC, tet*(M), *vanB1, trans, trans1*, IS*150*	*efaA_fm_, acm*	*–*	1, 2, 14	B	18	7	1	1	1	5	1	1
CCRI-16717	H	Stools	CIP, ERY, GEN, KAN, LIN, AMP, NIT, STR, TET, TYL, VAN	*aac(6′)-Ii, aadE, aph(3′)-IIIa, erm*(AM), *erm*(B), *sat(4), msrC, tet*(M), *tet*(L), *vanA, vanH, vanR, vanS, vanX, vanY, res, trans*, IS*1182*	*efaA_fm_, acm*	*–*	1, 2, 4, 6, 11	D	1	8	4	5	7	1	1	5
CCRI-18707	H	Pus	CIP, ERY, GEN, KAN, LIN, AMP, PEN, STR, TYL	*aac(6′)-Ii, aac(6′)-Ie-aph(2″)-Ia, aadE, aph(3′)-IIIa, erm*(AM), *sat(4), erm*(B), *msrC, trans, trans1*, IS*1182*, IS*150*	*efaA_fm_, esp, acm, hyl*	*+*	17, Unique	C	65	1	2	1	20	1	1	9
CCRI-18231	H	Wound	CIP, ERY, GEN, KAN, LIN, AMP, PEN, STR, TYL	*aac(6′)-Ii, aac(6′)-Ie-aph(2″)-Ia, aadE, aph(3′)-IIIa, erm*(AM), *erm*(B), *sat(4), msrC, trans, trans1*, IS*1182*, IS*150*	*efaA_fm_, esp, acm, hyl*	*+++*	17, Unique	G	205	3	1	1	1	1	1	1

a *D, dogs; H, humans*.

b *AMP, ampicillin; CHL, chloramphenicol; CIP, ciprofloxacin; ERY, erythromycin; GEN, gentamicin; KAN, kanamycin; LIN, lincomycin; NIT, nitrofurantoin; PEN, penicillin; STR, streptomycin; TET, tetracycline; TYL, tylosin; VAN, vancomycin*.

c *Identified by microarray; aac(6′)-Ii is intrinsic resistance in E. faecium*.

Weak biofilm producer (+) and strong biofilm producer (+++), based upon the previously calculated OD values (see Materials and Methods)

### Antibiotic susceptibility testing

Isolates were tested for MIC using the Sensititre plate CMV3AGPF (Trek™ Diagnostic System Ltd, Cleveland, OH) according to the Clinical and Laboratory Standards Institute (CLSI, M31-A3 and M100-S20) guidelines (Clinical and Laboratory Standards Institute, 2008, 2010). Ciprofloxacin and ampicillin susceptibilities were further analyzed by standard broth macrodilution method (CLSI, M31-A3 and M100-S20). Breakpoints from CLSI and the Canadian Integrated Program for Antimicrobial Resistance Surveillance (CIPARS) were used (Anonymous, 2008). *Staphylococcus aureus* ATCC29213 and *Enterococcus faecalis* ATCC29212 were used as control strains.

### PFGE and MLST

Isolates were analyzed for clonal diversity by pulsed-field gel electrophoresis (PFGE) after *Sma*I (New England Biolabs, Inc., Beverly, Ma, USA) digestion as described by Garcia-Migura et al. (2005). Digested DNA was electrophoresed as previously described (Vankerckhoven et al., 2008). Computer analysis of PFGE banding patterns was performed with Bionumerics Version 6.5 software (Applied Maths, Austin, TX, USA). Banding pattern similarities were analyzed by the Dice coefficient, and cluster analysis was performed by the unweighted-pair group method using average linkages (UPGMA). PFGE types were determined as =80% similarity. MLST was based on seven *E. faecium* housekeeping genes (*atpA, ddl, gdh, purk, gyd, pstS*, and *adk*). Different sequences were assigned allele numbers, and different allelic profiles were assigned STs based on the MLST database (http://www.mlst.net/databases/). Analysis was performed using the eBURST version 3 algorithm implemented as a Java applet at http://eburst.mlst.net.

### DNA microarrays

Microarray hybridization experiments were performed as previously described (Champagne et al., 2011). This enterococcal virulence microarray was developed (Diarra et al., 2010) at the National Research Council in Montreal and carries 70 taxonomic probes for species identification as well as 15 virulence factors, and 173 antibiotic resistance probes for a total of 262 probes. The microarray was used for the detection of putative target genes. Briefly, bacterial DNA isolated from single colonies was labeled with Cy5-dCTP (GE Healthcare, Little Chalfont, UK). Hybridization, washing, scanning, image processing, scoring, and data analysis were done as previously described (Champagne et al., 2011). Arrays were scanned by a ScanArray Express microarray Scanner (Packard Biosciences, Billerica, MA, USA). Oligonucleotide spots with a signal-to-noise fluorescence ratio above 3 were considered positive. Positive microarray results were confirmed by PCR with specific primers for the following genes *tet*(M), *tet*(O), *tet*(L), *erm*(B), *agg* and *esp* (Table 2). Since *erm*(AM) and *erm*(B) shared over 80% similarity and are perfectly correlated, they were considered as the same in this study as previously proposed (Roberts et al., 1999).

**Table 2:** **Primers and conditions used in PCR for confirmation of microarray results and for *rep*-like genes identification**.

**Gene detected or Rep-family no.^a^**	**Primers (5′ to 3′)^b^**	**Amplicon (bp)**	**Annealing (T°C)**	**Reference or GenBank no.**
*tet*(M)	F-GTGGACAAAGGTACAACGAG	406	55	De Leener et al., 2004
	R-CGGTAAAGTTCGTCACACAC			
tet(*O*)	F-GATGGCATACAGGCACAGAC	614	62	Aarestrup, 2000
	R-CAATATCACCAGAGCAGGCT			
*tet*(L)	F-ATAAATTGTTTCGGGTCGGTAAT	1077	52	De Leener et al., 2004
	R-AACCAGCCAACTAATGACAAGT			
*erm*(B)	F-GAAAAGRTACTCAACCAAATA	639	52	Poeta et al., 2005
	R-AGTAACGGTACTTAAATTGTTTAC			
*agg*	F-GGTGCCACAATCAAATTAGG	380	46	Seno et al., 2005
	R-GATTCTTCGATTGTGTTGTAAACG			
*esp*	F-TTGCTAATGCTAGTCCACGACC	955	63	Seno et al., 2005
	R-GCGTCAACACTTGCATTGCCGAA			
1 (pIP501)	F-TCGCTCAATCACTACCAAGC	624	52	X17655
	R-CTTGAACGAGTAAAGCCCTT			
9 (pCF10)	F-GCTCGATCARTTTTCAGAAG	201		AY855841
	R-CGCAAACATTTGTCWATTTCTT			
4 (pMBB1)	F-ACTATGTCGTTGAGTCTAATGACT	430	52	U26268
	R-AGCAAGATAGAATATTTACTTTTAAGTTT			
14 (pRI1)	F-RTTTTGRCTTTCTTSYTTCA	164		EU327398
	R-TGAAAGYTTRGATAGYTTTGC			
17 (pRUM)	F-TACTAACTGTTGGTAATTCGTTAAAT	502		EU376117
	R-ATCAAGGACTCAACCGTAATT			
Unique (pMG1/pHTβ)	F-GTATTAACACACTGGACTC	199	52	AB206333
	R-TCAGTGTAGGCAATAACCC			
2 (pRE25)	F-GAGAACCATCAAGGCGAAAT	630	56	X92945
	R-ACCAGAATAAGCACTACGTACAATCT			
6 (pS86)	F-ACGAATGAAAGATAAAGGAGTAG	551		AJ223161
	R-TAAATTCTAGTTTGGCAATCTTAT			
8 (pAM373)	F-CCAATCATGTAATGTTACAACC	394		AE002565
	R-TAGATACGACAAAAGAAGAATTACA			
18 (pEF418)	F-ACACCAGTCGAAATGAATTT	462		AF408195
	R-AGGAATATCAAGTAATTCATGAAAGT			
13 (pC194)	F-TACCAGAATAYTTAGCCATTTC	402	54	V01277
	R-ATGATGCAATATATTAAGCA			
15 (pUSA03)	F-CAGTAGAAGAAAATTATAAAGAAC	327		CP000258
	R-GTTATGGCTGGTTTTAATAAA			
16 (pSAS)	F-CTTCTATATCACTATCATTGTCATT	592		BX571858
	R-CAGGAAAACACTTCGTTTAT			
11 (pEF1071)	F-TCTAGAATGCGTGAAAAAGG	500	54	AF164559
	R-CCTTTGAAGATWGCRGTWAG			

a *Rep-family numbers are associated with plasmid in parenthesis*.

b *F, forward; R, reverse*.

### PCR detection of virulence and antibiotic resistance genes

PCR amplifications of virulence genes *acm* and *hyl* and of the quinolone resistance-determining regions (QRDR) genes were performed as previously described (Nallapareddy et al., 2003; Vankerckhoven et al., 2004; Billstrom et al., 2008; Werner et al., 2010). The N-terminal and C-terminal regions of the *pbp5* gene were amplified by PCR (Aarestrup et al., 2002; Poeta et al., 2007). Both strands of the purified QRDRs and *pbp5* amplicon products were sequenced. Sequences of *gyrA/B* and *parC/E* were compared with the *E. faecium* DO genome (GenBank accession no. CP003583) whereas sequences of the C- and N-terminal regions of *pbp5* gene were compared with *pbp5* gene reference sequence (GenBank accession no. X84860).

### PCR for *repa* genes (plasmid families)

All isolates were screened for *rep*-like sequences by PCR as previously described (Jensen et al., 2010) with few modifications (Table 2). Briefly, fourteen different *rep*-family plasmids (*rep*_1–2_, *rep*_4_, *rep*_6_, *rep*_8–9_, *rep*_11_, *rep*_13–15_, *rep*_16–18_ and *rep*_*Unique*_) were tested for their presence in *E. faecium* isolates. The PCR amplified and sequenced amplicons from selected strains (*E. faecium* strain no. 07-5598, M2146-08, 18581 and 16717) from this study were used as positive controls for *rep*_1–2_, *rep*_4_, *rep*_6_, *rep*_14_, *rep*_17–18_ and *rep*_*Unique*_ whereas control strains for *rep*_8–9_, *rep*_11_, *rep*_13_, and *rep*_15–16_ were from a previous study (Tremblay et al., 2012). The families' *rep*_3_, *rep*_5_, *rep*_7_, *rep*_10_, and *rep*_19_ could not be tested because no positive PCR products were obtained and no control strains were available.

### Detection of CRISPR-*cas*

The CRISPR1-*cas* and CRISPR3-*cas* loci were screened by PCR as previously described (Palmer and Gilmore, 2010) with slight modifications. Briefly, the PCR reactions were performed in a total of 50 μl, using 80 pmol of each primer, 1.5 mM MgCl_2_, 10 mM each of dNTPs, 2 U of Taq DNA, 1X Buffer mix (New England Biolabs) and sample DNA. Amplification reactions were carried out using a Whatman Biometra thermocycler (Montreal Biotech Inc, Québec, Canada) programmed as follows: an initial denaturation step of 94°C for 2 min, 30 cycles of denaturation at 94°C for 1 min, annealing for 1 min and extension at 72°C for 1 min, followed by a final elongation at 72°C for 5 min. For visualization, 5 μl of the PCR reaction were subjected to electrophoresis in 1.2% agarose gel stained with ethidium bromide. A 100 bp ladder (TrackIt, *Invitrogen*, Ontario, Canada) was used as a marker. *Enterococcus faecalis* strain no. 02-A701 and 06-6225 were used as positive controls.

### Biofilm formation

Isolates were inoculated in tryptic soy broth (TSB) supplemented with 1% glucose in 96 well microtiter plates (Fisher scientific) for bacterial growth and biofilm formation as described elsewhere (Zoletti et al., 2011). Biofilm was quantified using crystal violet staining method as described by Zoletti et al. (2011). *Staphylococcus epidermidis* ATCC 35984 (biofilm producer) and wells containing uninoculated medium were used as controls. ODs were obtained using a microplate reader Biotek Synergy HT (Bio-tek Instruments Inc., Winooski, VT, USA) at a wavelength of 570 nm. Analyses were based on two different experiments where isolates were tested in triplicate. The quantification of biofilm formation in microtiter plates was performed as previously described (Stepanovic et al., 2000, 2007). Briefly, strains were divided into the following categories: no biofilm producer (−), weak biofilm producer (+), moderate biofilm producer (++) and strong biofilm producer (+++), based upon the previously calculated OD values: OD ≤ ODc = no biofilm producer; ODc < OD ≤ 2X ODc = weak biofilm producer; 2X ODc < OD ≤ 4X ODc = moderate biofilm producer; 4X ODc < OD = strong biofilm producer. ODc is defined as three standard deviations (SD) above the mean OD of the negative control.

### Statistical analysis

A chi-square exact test was used to examine the association between isolate, origin, antibiotic resistance, virulence genes, biofilm formation, plasmid families and CRISPR genes. Statistical analyses were carried out using SAS software v. 9.2 (SAS Institute Inc., Cary, N.C., USA). The level of statistical significance was set at 0.05.

## Results

### MLST and PFGE

MLST allelic profiles are presented in Table 1. A total of six STs were found among the *E. faecium* isolates, ST1 (*n* = 1), ST18 (*n* = 2), ST65 (*n* = 1), ST202 (*n* = 2), ST205 (*n* = 1) and one novel, ST803 (isolates M2146-08, M5853-09 and M20638-11) (Table 1). Canine ARE belonged to ST202 (*n* = 2) and ST803 (*n* = 3) both SLV of ST17. Human clinical isolates belonged to ST65 (*n* = 1), a singleton known to be found only among clinical strains (Werner et al., 2011), and ST205 (*n* = 1), a SLV of ST17 (Table 1). Human surveillance isolates belonged to ST18 (*n* = 2), and ST1 (*n* = 1, linked to CC1). Therefore, all these STs were linked to HA-ARE except for ST1 which was shown to belong to a cluster containing primarily isolates from calves in the Netherlands (http://efaecium.mlst.net/). Following genomic *Sma*I digestion and PFGE, the *E. faecium* isolates (*n* = 10) of human and canine origins produced ten macro-restriction patterns clustered into eight PFGE types, termed A through H (Table 1). Canine *E. faecium* isolates of subtypes A and E were ≥ 80% similar whereas the remaining *E. faecium* isolates were considered unrelated (≤80%). The *E. faecium* PFGE types B and H were set as a same group by MLST (ST18) whereas the new ST803 was grouped as PFGE types E and F (Table 1).

### Antibiotic resistance

Chloramphenicol, ciprofloxacin, erythromycin, lincomycin, tylosin, gentamicin, kanamycin, streptomycin, penicillin, ampicillin, and tetracycline resistances were observed in *E. faecium* clinical isolates of canine origin with no resistance observed to daptomycin, linezolid, nitrofurantoin, quinupristin/dalfopristin, vancomycin and tigecycline (Tables 1, 3). Resistances to ciprofloxacin, erythromycin, lincomycin, tylosin, gentamicin, kanamycin, streptomycin, nitrofurantoin, penicillin, ampicillin, tetracycline and vancomycin but not to chloramphenicol, daptomycin, linezolid, quinupristin/dalfopristin and tigecycline were observed in human *E. faecium* clinical isolates (Tables 1, 3). All *E. faecium* isolates were resistant to ampicillin, ciprofloxacin and lincomycin. Seven isolates were considered as high-level ciprofloxacin-resistant (MICs of > 16 μg/ml) whereas nine isolates showed high-level ampicillin resistance (MICs of ≥ 256 μg/ml) (Table 3). Also, erythromycin resistance (*n* = 6) was positively associated (*p*-value < 0.05) with aminoglycoside resistance (*n* = 6), whereas ciprofloxacin resistance (*n* = 10) correlated (*p*-value < 0.05) with ampicillin (*n* = 9) resistances. All vancomycin resistant *E. faecium* (*n* = 3) isolates were of human origin.

**Table 3:**
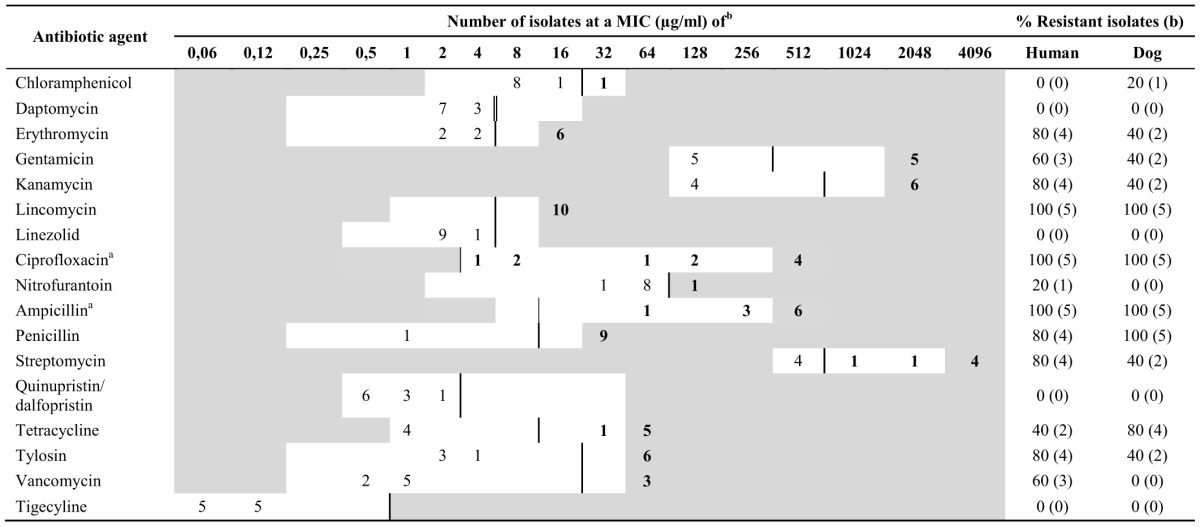
**Antibiotic resistance of *E. faecium* clinical isolates from humans and dogs based on MICs**.

*^a^Ciprofloxacin and ampicillin MICs from the standard broth macrodilution method*.

*^b^Number of isolates; |, MIC breakpoint; bold numbers, numbers of resistant isolates; ‖, susceptible breakpoint; unshaded areas, range of dilutions tested; numbers in the shaded area, isolates with MICs greater than the highest tested concentrations*.

### Microarray and PCR analysis of virulence and antibiotic resistance genes

Virulence and antibiotic resistant genotypes are presented in Table 1. Results demonstrated all canine ARE isolates were *acm*-positive but were negative for *esp* and *hyl* genes. The majority of these isolates were shown to harbor *efa*A_*fm*_ gene, encoding for a cell wall adhesin. One canine ARE isolate also had the *agg* gene encoding for an aggregation substance of pheromone-responsive plasmid. The *efa*A_*fm*_ gene was detected in all human isolates but one. The two human clinical isolates (CCRI-18707 and -18231) were also positive for the glycosyl hydrolase gene (*hyl*). Acquisition of multiple antibiotic resistance genes was observed ranging from 2 to 14 per isolate. Correlation between MICs and the presence of resistance genes has indicated that tetracycline resistance was attributable to *tet*(M) (*n* = 2); *tet*(M) and *tet*(L) (*n* = 3); and *tet*(M), *tet*(L) and *tet*(O) (*n* = 2). Resistance to erythromycin, lincomycin and tylosin could be explained by the presence of *msrC*, *erm*(AM)/ *erm*(B) in all strains with the exception of one (07-5598). Streptomycin resistance was associated with the *aadE* gene in all strains except one (07-5598). Kanamycin resistance was explained by *aph(3′)-IIIa* in all strains except two (07-5598 and M2971-08) whereas gentamicin resistance was attributable to *aac(6′)-Ie-aph(2′)-Ia* in all strains but two (07-5598 and CCRI-16717). Chloramphenicol resistance (*n* = 1) could not be explained because both *cat* and *floR* genes were absent in the two canine ARE resistant isolates. The gene *aac(6′)-Ii* is intrinsic in *E. faecium* and confers resistance to low-level aminoglycosides. Also, the transposase genes *trans* and *trans1* (both GenBank accession no. AF516335) associated with transposons Tn*1546* and Tn*5405-like* on the plasmid pUW786 carrying multiresistance gene cluster were identified in many isolates (*n* = 9). A correlation (*p*-value < 0.05) was observed between *aadE* (high-level streptomycin resistance), *aph(3′)-IIIa*, (aminoglycoside resistance), *sat(4)* (streptothricin acetyltransferase) and *IS1182* transposase gene (also associated with plasmid pUW786) in human isolates of *E. faecium* (Diarra et al., 2010). The antibiotic resistance genes *erm*(AM)/*erm*(B) (macrolide-lincosamide-streptogramin B, MLS_*b*_), *aadE*, *sat(4)*, *aph(3′)-IIIa* were significantly (*p*-value < 0.05) associated with human isolates. Positive associations (*p*-value < 0.05) were observed between *trans1* and *IS150* transposase genes, *aac(6′)-Ie-aph(2″)-Ia* (encoding for a bi-functional aminoglycoside-modifying enzyme) and *erm*(AM)/*erm*(B). No beta-lactamase genes were detected in this study.

### Screening for *gyrA/B* and *ParC/E* mutations

*Enterococcus faecium* isolates spanned a range of ciprofloxacin MICs from 4 μg/ml to > 256 μg/ml with seven isolates considered as high-level ciprofloxacin-resistant (MICs of > 16 μg/ml) (Table 3). High-level ciprofloxacin resistance was attributable to ParC (Ser80 → Arg, Ile or Asp; and Asn73 → Asp) and GyrA (Ser83 → Arg, Tyr, or Ile; Met127 → Trp; and Glu87 → Lys) mutations (Table 4). All high-level ciprofloxacin-resistant isolates had mutations in both GyrA (Ser83 → Arg or Tyr or Ile) and ParC (Ser80 → Arg or Ile or Asp). Corresponding fragments of subunits B (*parE/gyrB*) were also investigated. Two isolates demonstrated mutations in GyrB (Asp436 → Asn, Leu371 → Trp and Pro455 → Ser) whereas no changes in ParE were detected (Table 4). Isolates with MICs of < 16 μg/ml did not harbor mutational changes.

**Table 4:** **Mutations in *gyrA/B* and *parC* of clinical *E. faecium* isolates with their corresponding ciprofloxacin MIC**.

**Ciprofloxacin MIC (μg/ml)**	**Isolate no.**	**Host^a^**	**Amino acid^b^ mutation in gene (codon)**		
			***gyrA***	***gyrB***	***parC***
4	M2146-08	D	None	None	None
8	M5853-09	D	None	None	None
8	CCRI-16717	H	None	None	None
64	M20638-11	D	I (83)	N (436)	R (80)
128	07-5598	D	Y (83), W (127)	None	I (80)
128	CCRI-18231	H	K (87)	None	I (80), D (73)
>256	M2971-08	D	Y (83)	None	I (80)
>256	CCRI-18581	H	Y (83)	None	R (80)
>256	CCRI-16354	H	Y (83)	W (371), S (455)	I (80)
>256	CCRI-18707	H	R (83)	None	I (80)

a *D, dogs; H, humans*.

b *D, aspartic acid; I, isoleucine; K, lysine; N, asparagine; R, arginine; S, serine; W, tryptophan; Y, tyrosine*.

### Sequence analysis of *pbp5*

Nine isolates showed high-level ampicillin resistance (MICs of ≥ 256 μg/ml) with all canine ARE being high-level. Both the N-terminal and C-terminal regions were analyzed and revealed that PBP5 contained 25 amino acid changes, as shown in Table 5. The insertion of aspartic acid at position 466′ was mostly observed in isolates of animal origin. Alleles were designated 1-5 based on important amino acid substitutions in the C-terminal region with none showing 100% identity with the reference sequence (GenBank accession no. X84860). The same *pbp5* alleles (alleles 1 and 2) were observed in clonal isolates with PFGE patterns A1 and A2 and E1 and E2.

**Table 5:** **Amino acid changes detected in the C- and N-terminal regions of *pbp5* of clinical *E. faecium* isolates**.

**MIC ampicillin (μg/ml)**	**Host^a^**	***E. faecium***	**Amino acid^b^ change at position^c^**																								***pbp5* allele^d^**
			**N-terminal**											**C-terminal**													
			**66**	**68**	**85**	**100**	**144**	**172**	**177**	**204**	**216**	**324**	**361**	**461**	**466′**	**470**	**476**	**485**	**496**	**497**	**499**	**525**	**586**	**615**	**629**	**643**	
−	−	X84860	G	A	E	E	K	T	L	D	A	T	G	Q	−	H	A	M	N	F	A	E	V	A	E	D	−
256	D	07-5598	NA	**T**	**D**	**Q**	**Q**	**A**	**I**	**G**	**S**	**A**	NA	Q	**D**	**Q**	**S**	M	**K**	**L**	**T**	**D**	V	A	**V**	D	1
>256	D	M2971-08	NA	**T**	**D**	**Q**	**Q**	**A**	**I**	**G**	**S**	**A**	NA	Q	**D**	**Q**	**S**	M	**K**	**L**	**T**	**D**	V	A	**V**	D	1
>256	H	CCRI-18581	NA	**T**	**D**	**Q**	**Q**	**A**	**I**	**G**	**S**	**A**	**E**	Q	**D**	**Q**	**S**	M	**K**	**L**	**T**	**D**	V	A	**V**	NA	1
>256	D	M2146-08	**E**	**T**	**D**	**Q**	**Q**	**A**	**I**	**G**	**S**	**A**	NA	Q	**D**	**Q**	A	**A**	**K**	F	**T**	**D**	**L**	A	**V**	**E**	2
>256	D	M5853-09	**E**	**T**	**D**	**Q**	**Q**	**A**	**I**	**G**	**S**	**A**	NA	Q	**D**	**Q**	A	**A**	**K**	F	**T**	**D**	**L**	A	**V**	D	2
>256	D	M20638-11	NA	**T**	**D**	**Q**	**Q**	**A**	**I**	**G**	**S**	**A**	G	Q	**D**	**Q**	A	**A**	**K**	F	**T**	**D**	**L**	A	**V**	D	2
64	H	CCRI-16717	NA	NA	E	**Q**	**Q**	**A**	**I**	D	**S**	**A**	**E**	Q	−	**Q**	A	M	**K**	F	**I**	**D**	V	A	E	D	3
>256	H	CCRI-16354	**E**	**T**	**D**	**Q**	**Q**	**A**	**I**	**G**	**S**	**A**	NA	**K**	−	**Q**	A	**A**	**K**	F	**T**	**D**	**L**	A	**V**	D	4
256	H	CCRI-18707	**E**	**T**	**D**	**Q**	**Q**	**A**	**I**	**G**	**S**	**A**	**E**	**K**	−	**Q**	A	**A**	**K**	F	**T**	**D**	**L**	A	**V**	NA	4
256	H	CCRI-18231	NA	**T**	**D**	**Q**	**Q**	**A**	**I**	**G**	**S**	**A**	NA	Q	−	**Q**	A	**T**	**K**	F	**T**	**D**	**L**	**S**	**V**	D	5

*NA, The sequence at this position was not available*.

a *D, dogs; H, humans*.

b *A, alanine; D, aspartic acid; E, glutamic acid; F, phenylalanine; G, glycine; H, histidine; K, lysine; L, leucine; M, methionine; N, asparagine; Q, glutamine; S, serine; T, threonine; V, valine*.

c *Amino acid changes with respect to the reference sequence (GenBank accession no. X84860) are indicated in bold*.

d *Alleles were designated 1–5 based on important amino acid substitutions in the C-terminal region*.

### *Repa* genes (plasmid families)

Nine different *rep*-family plasmid genes (*rep*_1–2_, *rep*_4_, *rep*_6_, *rep*_11_, *rep*_14_, *rep*_17–18_ and *rep*_*Unique*_) were detected in *E. faecium* isolates (Table 1). The predominant *rep*-family among *E. faecium* isolates was *rep*_11_ (pEF1071) with seven isolates. Other *rep*-families were also observed: *rep*_1_ (pIP501) (*n* = 2), *rep*_2_ (pRE25) (*n* = 6), *rep*_4_ (pMBB1) (*n* = 1), *rep*_6_ (pS86) (*n* = 5), *rep*_14_ (pRI1) (*n* = 5), *rep*_17_ (pRUM) (*n* = 3), *rep*_18_ (pEF418) (*n* = 1) and *rep*_*Unique*_ (pUB101) (*n* = 3). Overall, *rep*_11_ and *rep*_6_ families were significantly (*p*-value < 0.05) associated with isolates of canine origin. The families' *rep*_8–9_, *rep*_13_, and *rep*_15–16_ were not detected.

### CRISPR-*cas* genes

To determine whether there is an association between CRISPR elements and plasmid family genes, virulence and/or antibiotic resistance genes, CRISPR-*cas* genes were investigated by PCR. Because potential sequence divergence among *csn1* genes, an internal region of CRISPR-*cas* locus-specific genes, may lead to false-negative PCR results, isolates with negative PCR results were further screened with primers flanking the conserved locations of the CRISPR1-*cas* and CRISPR3-*cas* loci, between homologues of EF0672-EF0673 and EF1760-EF1759, respectively, as compared with the *E. faecalis* genome V583 (Palmer and Gilmore, 2010). According to Palmer and Gilmore (2010), one CRISPR-*cas* locus was identified in three *E. faecium* genomes. However, this locus could not be detected in our ten ARE strains. No significant correlations could be made with the absence of antibiotic resistance, virulence, and plasmid family genes due to low number of isolates.

### Biofilm formation

Both isolates producing biofilm were recovered from infections, one weak and one strong both harboring the *esp* gene (Table 1), contrasting with those from surveillance. However, one ARE isolate which colonized a hospitalized patient was positive for the *esp* gene and did not produce a biofilm.

## Discussion

To the best of our knowledge, we describe for the first time, the characterization of ARE strains of canine origin from Canada with STs identical (ST202) or closely related (ST803) to human hospital-associated lineages. Colonization by major clones is relatively rare in healthy humans (De Regt et al., 2012). The presence in canine clinical cases of identical or closely related ARE clones currently involved in the nosocomial epidemiology supports the hypothesis that, cross-transmission between humans and dogs may potentially occur. This is also why it was decided to fully characterized these dog strains and compare them with human strains from Canada. In canines, reports have indicated that ARE most frequently were of ST266 origin but that a variety of STs associated with human clinical infections could also be found (Damborg et al., 2008, 2009; Ghosh et al., 2011; De Regt et al., 2012). These findings, along with the current study, present evidence of some genotypic concordance, based on MLST, between hospital clones of human origin and community ARE from clinical cases of dogs, indicating that these isolates are likely evolutionarily linked. This has also been previously suggested by a phylogenomic analysis of two dog strains and seven sequenced *E. faecium* genomes derived from humans (De Regt et al., 2012). Similar to a study on Dutch canine ARE isolates (De Regt et al., 2012), canine ARE isolates in our study were not an “exact copy” to those in the circulating Canadian hospital reservoir as none of the human predominant types in Canada (McCracken et al., 2013) were identified in these isolates. This could also be because the number of isolates of this study was limited.

In humans, the emergence of high-level ampicillin resistance, specifically in US hospitals in the early 1980s, preceded the epidemic rise of vancomycin resistance, which occurred in the 1990s, explaining why virtually all vancomycin-resistant enterococci (VRE) recovered from nosocomial infections in humans are also ampicillin resistant (Grayson et al., 1991; Iwen et al., 1997). In this study, the transposase genes *trans* and *trans1*, associated with Tn*1546*, were present in strains of canine origin which were all susceptible to vancomycin. This could be explained by possible mutations or deletions within this transposon causing the susceptible phenotype in canine isolates. Because the association between ampicillin and vancomycin resistance phenotypes in humans probably reflects sequential and independent acquisition of resistance genes resulting in the selective dominance of a small subset of hospital-adapted clones (Willems et al., 2011), care should be taken to completly withdraw vancomycin from veterinary medicine and to perform detailed surveillance studies on possible anthropo-zoonotic transfer of ARE.

In this study, the high-level ciprofloxacin and ampicillin resistances observed were associated respectively with previously reported amino acid changes in topoisomerase IV, DNA gyrase and in PBP5 (Ligozzi et al., 1996; Zorzi et al., 1996; Rice et al., 2001; Jureen et al., 2003; Werner et al., 2010). To our knowledge, previous studies did not observe mutations in the *gyrB* target (Leavis et al., 2006; Werner et al., 2010; Valdezate et al., 2012). The contribution of these amino acid changes in GyrB to ciprofloxacin resistance is difficult to evaluate because amino acid changes in GyrA and ParC alone are sufficient to confer high-level resistance to ciprofloxacin. Other resistance mechanisms may be responsible for (low-level) ciprofloxacin resistance in HA ARE isolates that do not contain amino acid changes in the QRDRs of ParC and GyrA. Recently, characterization of EfmA, a multidrug efflux pump conferring resistance to quinolones and macrolides, from *E. faecium* has been described (Nishioka et al., 2009) which could explain low-level ciprofloxacin resistance in some of the isolates of this study.

So far, canine ARE have been shown to carry fewer virulence traits (De Regt et al., 2012; Kwon et al., 2012) compared to human HA ARE (Top et al., 2008; Werner et al., 2010). It was also shown in a recent report that canine *E. faecium* did not form biofilms and lacked the strong gelatinase phenotype as well as *esp* (Ghosh et al., 2011). Also, as observed in our study, ARE strains that colonize the gastrointestinal tract of humans have been shown to carry less determinants such as the enterococcal surface protein, Esp (Coque et al., 2002; Heikens et al., 2007), genomic islands (Heikens et al., 2008), and insertion sequence elements (Leavis et al., 2007). Recently, a sequence-based classification system for enterococcal plasmids has been established which targets replicon-specific plasmid DNA to determine 19 plasmid families which are associated with either a very narrow or a broader host range (Jensen et al., 2010). This procedure was attempted to determine whether specific plasmid families were involved in ARE of canine origin vs. human origin. In this study, *rep*_6_ (small cryptic plasmids) and *rep*_11_ (toxin producing plasmids) plasmid families were significantly associated with isolates from dogs. Interestingly, it was also observed that these two families seem to be associated with human isolates from colonization (CCRI no. 18581, 16717, and 16354) but due to low number of isolates this statistical significance could not be addressed. No significant association between *rep* families and antimicrobial resistance genes could be identified for isolates of canine origin whereas the human clinical strains were significantly associated (*p* < 0.05) with *erm*(B)/*erm*(AM), *aadE, sat(4)*, *aph(3′)-IIIa*, IS*1182* and with the *rep*_17_ plasmid family which has been previously reported (Grady and Hayes, 2003). Interestingly, these genes were also detected in a surveillance human isolate. Recently, a significant correlation between the absence of CRISPR-*cas* loci and the presence of antibiotic-resistance genes was previously described for *E. faecalis* (Palmer and Gilmore, 2010). This tendency was also observed in ARE of this study but due to the low number of isolates its significance could not be determined. Further research is needed to assess the virulence and the antimicrobial resistance of canine strains in comparison with that of human strains and, more generally, to quantify the magnitude of this possible emerging zoonotic problem.

In conclusion, the current study provides the first characterization of canine clinical ARE isolates in Canada with STs identical or closely related to human clinical isolates. This analysis, as well as previous reports, further indicates that canine ARE isolates are evolutionarily linked with hospital ARE isolates with some discordance in their multi-drug resistance and virulence attributes. These findings also support the importance of prudent use of antibiotics in veterinary medicine to avoid zoonotic spread and development of vancomycin resistance of canine ARE isolates. Select and spread of HA-strains that can cause human infections is a major concern, independently of being VRE, because in some countries ARE associated with human infections remain vancomycin susceptible. Further studies are needed to understand the significance of dogs in the spread of this nosocomial pathogen in the community. The occurrence of ARE in dogs and other domestic animals could be addressed by national programs in order to explore the importance of the animal reservoir in the evolution of human hospital ARE isolates.

### Conflict of interest statement

The authors declare that the research was conducted in the absence of any commercial or financial relationships that could be construed as a potential conflict of interest.
